# Characterizing the Clinical Features and Atrophy Patterns of *MAPT*-Related Frontotemporal Dementia With Disease Progression Modeling

**DOI:** 10.1212/WNL.0000000000012410

**Published:** 2021-08-31

**Authors:** Alexandra L. Young, Martina Bocchetta, Lucy L. Russell, Rhian S. Convery, Georgia Peakman, Emily Todd, David M. Cash, Caroline V. Greaves, John van Swieten, Lize Jiskoot, Harro Seelaar, Fermin Moreno, Raquel Sanchez-Valle, Barbara Borroni, Robert Laforce, Mario Masellis, Maria Carmela Tartaglia, Caroline Graff, Daniela Galimberti, James B. Rowe, Elizabeth Finger, Matthis Synofzik, Rik Vandenberghe, Alexandre de Mendonça, Fabrizio Tagliavini, Isabel Santana, Simon Ducharme, Chris Butler, Alex Gerhard, Johannes Levin, Adrian Danek, Markus Otto, Sandro Sorbi, Steven C.R. Williams, Daniel C. Alexander, Jonathan D. Rohrer, on behalf of the Genetic FTD Initiative (GENFI)

**Affiliations:** From the Department of Neuroimaging (A.L.Y., S.C.R.W.), Institute of Psychiatry, Psychology and Neuroscience, King's College London; Departments of Computer Science (A.L.Y., D.C.A.) and Medical Physics and Biomedical Engineering (D.M.C.), Centre for Medical Image Computing, University College London; Dementia Research Centre (M.B., L.L.R., R.S.C., G.P., E.T., D.M.C., C.V.G., L.J., J.D.R.), Department of Neurodegenerative Disease, UCL Queen Square Institute of Neurology, London, UK; Department of Neurology (J.v.S., L.J., H.S.), Erasmus Medical Centre, Rotterdam, the Netherlands; Cognitive Disorders Unit (F.M.), Department of Neurology, Donostia University Hospital; Neuroscience Area (F.M.), Biodonostia Health Research Institute, San Sebastian, Gipuzkoa, Spain; Alzheimer's Disease and Other Cognitive Disorders Unit (R.S.-V.), Neurology Service, Hospital Clínic, Institut d’Investigacións Biomèdiques August Pi I Sunyer, University of Barcelona, Spain; Neurology Unit (B.B.), Department of Clinical and Experimental Sciences, University of Brescia, Italy; Clinique Interdisciplinaire de Mémoire, Département des Sciences Neurologiques, CHU de Québec, and Faculté de Médecine (R.L.), Université Laval, Québec; Sunnybrook Health Sciences Centre, Sunnybrook Research Institute (M.M.), and Tanz Centre for Research in Neurodegenerative Diseases (M.C.T.), University of Toronto, Canada; Center for Alzheimer Research (C.G.), Division of Neurogeriatrics, Department of Neurobiology, Care Sciences and Society, Bioclinicum, Karolinska Institutet; Unit for Hereditary Dementias (C.G.), Theme Aging, Karolinska University Hospital, Solna, Sweden; Fondazione Ca’Granda (D.G.), IRCCS Ospedale Policlinico; University of Milan (D.G.), Centro Dino Ferrari, Italy; Department of Clinical Neurosciences and Cambridge University Hospitals NHS Trust (J.B.R.), University of Cambridge, UK; Department of Clinical Neurological Sciences (E.F.), University of Western Ontario, London, Canada; Department of Neurodegenerative Diseases (M.S.), Hertie-Institute for Clinical Brain Research and Center of Neurology, University of Tübingen; Center for Neurodegenerative Diseases (DZNE) (M.S.), Tübingen, Germany; Laboratory for Cognitive Neurology, Department of Neurosciences (R.V.), and Leuven Brain Institute (R.V.), KU Leuven; Neurology Service (R.V.), University Hospitals Leuven, Belgium; Faculty of Medicine (A.d.M.), University of Lisbon, Portugal; Fondazione IRCCS Istituto Neurologico Carlo Besta (F.T.), Milan, Italy; University Hospital of Coimbra (HUC), Neurology Service (I.S.), and Center for Neuroscience and Cell Biology (I.S.), Faculty of Medicine, University of Coimbra, Portugal; Department of Psychiatry, McGill University Health Centre (S.D.), and McConnell Brain Imaging Centre, Montreal Neurological Institute (S.D.), McGill University, Montreal, Canada; Nuffield Department of Clinical Neurosciences (C.B.), Medical Sciences Division, University of Oxford; Division of Neuroscience and Experimental Psychology (A.G.), Wolfson Molecular Imaging Centre, University of Manchester, UK; Departments of Geriatric Medicine and Nuclear Medicine (A.G.), University of Duisburg-Essen; Department of Neurology (J.L., A.D.), Ludwig-Maximilians Universität München; German Center for Neurodegenerative Diseases (DZNE) (J.L.); Munich Cluster of Systems Neurology (SyNergy) (J.L.), Munich; Department of Neurology (M.O.), University of Ulm, Germany; Departments of Neuroscience, Psychology, Drug Research, and Child Health (S.S.), University of Florence; and IRCCS Don Gnocchi (S.S.), Florence, Italy.

## Abstract

**Background and Objective:**

Mutations in the *MAPT* gene cause frontotemporal dementia (FTD). Most previous studies investigating the neuroanatomical signature of *MAPT* mutations have grouped all different mutations together and shown an association with focal atrophy of the temporal lobe. The variability in atrophy patterns between each particular *MAPT* mutation is less well-characterized. We aimed to investigate whether there were distinct groups of *MAPT* mutation carriers based on their neuroanatomical signature.

**Methods:**

We applied Subtype and Stage Inference (SuStaIn), an unsupervised machine learning technique that identifies groups of individuals with distinct progression patterns, to characterize patterns of regional atrophy in *MAPT-*associated FTD within the Genetic FTD Initiative (GENFI) cohort study.

**Results:**

Eighty-two *MAPT* mutation carriers were analyzed, the majority of whom had P301L, IVS10+16, or R406W mutations, along with 48 healthy noncarriers. SuStaIn identified 2 groups of *MAPT* mutation carriers with distinct atrophy patterns: a temporal subtype, in which atrophy was most prominent in the hippocampus, amygdala, temporal cortex, and insula; and a frontotemporal subtype, in which atrophy was more localized to the lateral temporal lobe and anterior insula, as well as the orbitofrontal and ventromedial prefrontal cortex and anterior cingulate. There was one-to-one mapping between IVS10+16 and R406W mutations and the temporal subtype and near one-to-one mapping between P301L mutations and the frontotemporal subtype. There were differences in clinical symptoms and neuropsychological test scores between subtypes: the temporal subtype was associated with amnestic symptoms, whereas the frontotemporal subtype was associated with executive dysfunction.

**Conclusion:**

Our results demonstrate that different *MAPT* mutations give rise to distinct atrophy patterns and clinical phenotype, providing insights into the underlying disease biology and potential utility for patient stratification in therapeutic trials.

Frontotemporal dementia (FTD) is a heterogeneous disorder characterized by behavioral and language difficulties. Approximately one-third of cases are inherited on an autosomal dominant basis, with the majority being due to mutations in progranulin (*GRN*), chromosome 9 open reading frame 72 (*C9orf72*), or microtubule-associated protein tau (*MAPT*).^1^ Previous studies have shown that the heterogeneity of FTD is in part related to distinct clinical features and atrophy patterns between these different genetic groups.^2,3^ However, there can also be substantial phenotypic heterogeneity within each genetic group.^4^

Although more than 70 *MAPT* mutations have been identified to date, only a few are common, with P301L, IVS10+16, and R406W being the most frequently described.^5^ Within-group pathologic heterogeneity in *MAPT* mutation carriers is related to the location of the mutation in the gene,^6^ and there is some evidence that phenotypic heterogeneity is similarly affected by the position of the mutation.^5,7^ However, studying the effect of specific mutations on disease phenotype is difficult because there are typically only a few individuals with each particular mutation. Here we took the reverse approach, in which we used an unsupervised learning technique—Subtype and Stage Inference (SuStaIn)^4^—to identify subgroups within *MAPT* mutation carriers with similar atrophy patterns. This enabled us to compare the *MAPT* mutations of individuals assigned to each subtype, providing greater statistical power than considering each mutation separately. Moreover, the SuStaIn subtypes account for heterogeneity in disease stage, improving the accuracy of the subtyping assignments^4^ by removing a key confound from the analysis and enabling subtyping of presymptomatic individuals. We further compared the clinical phenotypes of each subtype to gain insight into the relationship between *MAPT* mutation, atrophy pattern, and clinical presentation.

## Methods

### Participants

The Genetic FTD Initiative (GENFI) is a cohort study enrolling symptomatic carriers of mutations in the genes causing FTD as well as their adult (>age 18) at-risk first-degree relatives (i.e., both presymptomatic mutation carriers and people who are mutation-negative; i.e., noncarriers). For this study, all *MAPT* mutation carriers (82 total: 25 symptomatic, 57 presymptomatic) who had cross-sectional volumetric T1-weighted MRI data available from Data Freeze 4 of GENFI^2^ were selected for inclusion in our analysis. As a control population for *z* scoring imaging data, we used data from 300 noncarriers from the GENFI cohort with available cross-sectional volumetric MRI. As a control population for statistical testing, we used data from the 48 of these noncarriers who were first-degree relatives of known symptomatic carriers of mutations in the *MAPT* gene. Fifty of the 82 *MAPT* mutation carriers had follow-up MRI scans at 1 or more time points (total of 92 follow-up scans available), which were used to check the consistency of the SuStaIn subtype and stage assignments at follow-up.

### Standard Protocol Approvals, Registrations, and Patient Consents

Local ethics committees at each of the sites approved the study and all participants provided informed written consent.

### Imaging Data

The acquisition and postprocessing procedures have been described previously.^2^ Briefly, cortical and subcortical volumes were generated using a multiatlas segmentation propagation approach known as geodesic information flow (GIF)^8^ on T1-weighted MRI. The volumes of 19 cortical and 7 subcortical regions were calculated comprising the orbitofrontal cortex, dorsolateral prefrontal cortex, ventromedial prefrontal cortex, motor cortex, opercular cortex, frontal pole, medial temporal cortex, lateral temporal cortex, temporal pole, supratemporal cortex, medial parietal cortex, lateral parietal cortex, sensory cortex, occipital cortex, anterior cingulate cortex, middle cingulate cortex, posterior cingulate cortex, anterior insular cortex, posterior insular cortex, amygdala, hippocampus, caudate, putamen, nucleus accumbens, globus pallidus, and thalamus. The total cerebellar volume was also calculated. A list of the GIF subregions included in each cortical region is included in eTable 1 (doi.org/10.5061/dryad.rxwdbrv83). All volumes were corrected for head size (total intracranial volume calculated using SPM 12^9^), scanner field strength (1.5T or 3T), age, and sex by estimating a linear regression model in a control population of 300 noncarriers (see Methods: Participants) and then propagating this model to the *MAPT* mutation carriers. There were no significant differences in head size (*p* = 0.80, *t* test), field strength (*p* = 0.37, χ^2^ test), age (*p* = 0.56, *t* test), or sex (*p* = 0.35, χ^2^ test) between the *MAPT* mutation carriers and the control population, and the control population covered a wider age range than the mutation carriers. The corrected volumes were then converted into *z* scores relative to the control population for use as input to SuStaIn, giving the control population a mean of 0 and an SD of 1. As regional brain volumes decrease with disease progression, the *z* scores become negative as the disease progresses. For simplicity, we multiplied the *z* scores by −1, giving positive *z* scores that increase with disease progression.

### Genetic Data

Sequencing was performed at each site to determine the presence of the specific *MAPT* mutation. To avoid unblinding of genetic status (mutation carrier or noncarrier) for individuals from families with rare mutations, in the presymptomatic mutation carrier group we only report the individual mutations if there are also noncarriers with that particular mutation, or for individuals who converted to being symptomatic during follow-up.

### Clinical Data and Neuropsychology

All participants underwent the standard GENFI clinical and neuropsychological assessment.^2^ The GENFI clinical assessment includes noting the presence of behavioral, neuropsychiatric, language, cognitive, and motor symptoms on a scale similar to the Clinical Dementia Rating (CDR) instrument with 0 representing no symptoms, 0.5 questionable or very mild symptoms, and 1, 2, and 3 representing mild, moderate, and severe symptoms, respectively.^10^ The revised version of the Cambridge Behavioural Inventory (CBI-R) was also performed.^11^ The neuropsychological battery included the Wechsler Memory Scale–Revised Digit Span forward and backward (total score), the Trail Making Test (TMT) A and B (total time to complete and number of errors noted), Wechsler Adult Intelligence Scale–Revised Digit Symbol, Boston Naming Test (30-item modified version), verbal fluency (category and phonemic), and Wechsler Abbreviated Scale of Intelligence Block Design (total score).^2^

### Subtype and Stage Inference

SuStaIn was used to identify subgroups of *MAPT* mutation carriers with distinct progression patterns from cross-sectional imaging data.^4^ SuStaIn simultaneously clusters individuals into groups (subtypes) and reconstructs a disease progression pattern (set of stages) for each group using disease progression modeling techniques. Each progression pattern is described using a piecewise linear *z* score model, consisting of a series of stages where each stage corresponds to a biomarker (volume of a brain region) reaching a new *z* score. The optimal number of subtypes was determined using information criteria calculated through cross-validation^12^ to balance model complexity with internal model accuracy, as in reference 4. The subtype progression patterns identified by SuStaIn were visualized using BrainPainter.^13^

### Assigning Individuals to Subtypes and Stages

Individuals were subtyped by comparing the likelihood they belonged to each SuStaIn subtype (summing over SuStaIn stage) with the likelihood they were at SuStaIn stage 0 (i.e., had no imaging abnormalities). We called individuals with a higher probability of belonging to SuStaIn stage 0 than any of the SuStaIn subtypes “normal-appearing,” and individuals with a higher probability of belonging to a SuStaIn subtype than to SuStaIn stage 0 as “subtypable.” Each subtypable individual was then assigned to their most probable subtype. Individuals were staged by computing their average SuStaIn stage, weighted by the probability they belonged to each stage of each subtype.

### Statistical Analysis

We compared the demographics of participants assigned to each group (normal-appearing and each of the SuStaIn subtypes). To compare whether there were any differences between groups, we performed pairwise comparisons between groups using *t* tests for continuous variables and χ^2^ tests for categorical variables. We tested whether any mutations had a significantly different proportion of individuals assigned to each subtype by performing a χ^2^ test comparing the number of individuals assigned to each subtype for each mutation vs all the other mutations. We performed 2 sets of analyses to compare the clinical and neuropsychological test scores between individuals assigned to each of the SuStaIn subtypes. In the first set of analyses, we used Mann-Whitney *U* tests to perform pairwise comparisons between the subset of noncarriers who were relatives of individuals with *MAPT* mutations (n = 48) and symptomatic *MAPT* mutation carriers assigned to each SuStaIn subtype (n = 25 in total). In the second set of analyses, we accounted for SuStaIn stage, age, and sex, by fitting the linear model score ∼ subtype + stage + age + sex for each test, including data from all subtypable mutation carriers (n = 34; 9 presymptomatic and 25 symptomatic). We report statistical significance at a level of *p* < 0.05, and at the Bonferroni corrected level of *p* < 0.001 for the clinical scores (43 items), and *p* < 0.005 for the neuropsychology scores (11 items) to account for multiple comparisons.

### Data Availability

Data can be obtained according to the GENFI data sharing agreement, after review by the GENFI data access committee with final approval granted by the GENFI steering committee. Source code for the SuStaIn algorithm is available at github.com/ucl-pond/.

## Results

### Participant Demographics

Table 1 shows the demographics of the participants included in this study. SuStaIn was applied to 82 *MAPT* mutation carriers (25 symptomatic, 57 presymptomatic), consisting predominantly of individuals with P301L (n = 38), IVS10+16 (n = 20), and R406W (n = 9) mutations, but there were also additional rarer mutations, which are not fully disclosed to avoid unblinding of the genetic status. The large majority of symptomatic mutation carriers (23 out of 25) had a diagnosis of behavioral variant FTD, with 1 individual having a diagnosis of corticobasal syndrome, and another having a diagnosis of dementia that was not otherwise specified.

**Table 1:**
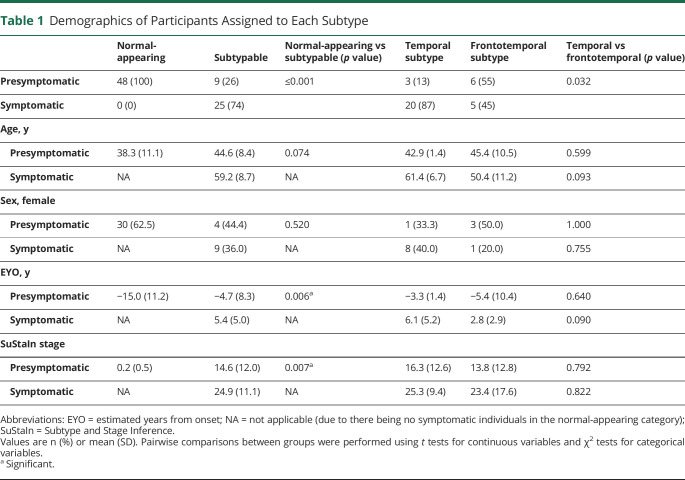
Demographics of Participants Assigned to Each Subtype

### Subtype Progression Patterns

**Figure 1:**
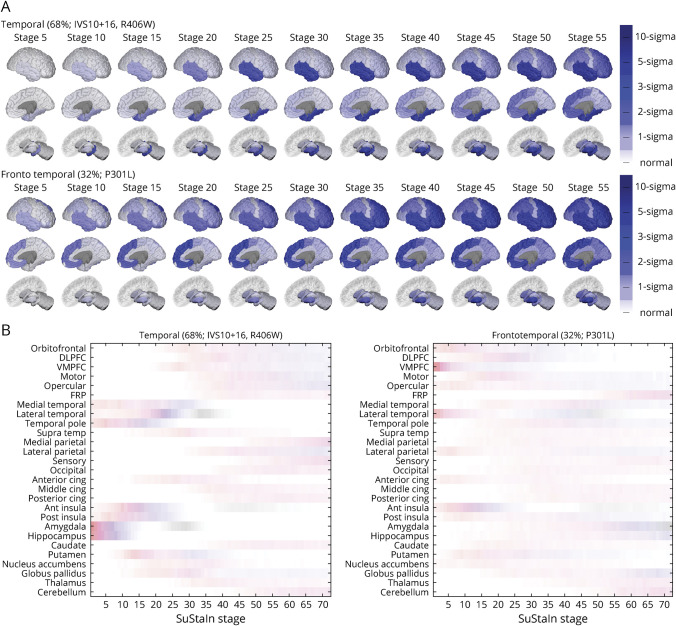
Subtype Progression Patterns Identified by Subtype and Stage Inference (SuStaIn) Each progression pattern consists of a set of stages at which regional brain volumes in *MAPT* mutation carriers (symptomatic and presymptomatic) reach different *z* scores relative to noncarriers. (A) Spatial distribution and severity of atrophy at each SuStaIn stage based on the most likely subtype progression patterns predicted by the SuStaIn algorithm. (B) Uncertainty in the SuStaIn subtype progression patterns for each region, where each region is shaded according to the probability a particular *z* score is reached at a particular SuStaIn stage, ranging from 0 (white) to 1 (red for a *z* score of 1, magenta for a *z* score of 2, blue for a *z* score of 3, and black for a *z* score of 5). Visualizations in subfigure A were generated using BrainPainter.^13^ Ant = anterior; Cing = cingulate; DLPFC = dorsolateral prefrontal cortex; FRP = frontal pole; Post = posterior; VMPFC = ventromedial prefrontal cortex.

SuStaIn identified 2 groups of *MAPT* mutation carriers with distinct patterns of regional atrophy (Figure 1). The first group, which we termed the “temporal subtype,” had atrophy in the hippocampus, amygdala, medial and lateral temporal cortex, and temporal pole as well as anterior and posterior insular cortex at early SuStaIn stages. The second group, which we termed the “frontotemporal subtype,” had atrophy in the orbitofrontal cortex, ventromedial prefrontal cortex, lateral temporal lobe, anterior insula cortex, and anterior cingulate at early SuStaIn stages. Thus, early atrophy in the anterior insula and lateral temporal lobe was a common feature of both subtypes; early atrophy in the medial temporal lobe, temporal pole, posterior insula, hippocampus, and amygdala was a distinctive feature of the temporal subtype; and early atrophy in frontal regions and the anterior cingulate was a distinctive feature of the frontotemporal subtype.

### Subtype Prevalence

Among the 25 symptomatic mutation carriers, 0 (0%) were categorized as normal-appearing (i.e., assigned to very early SuStaIn stages at which there is low confidence in the subtype assignment), 20 (80%) were assigned to the temporal subtype, and 5 (20%) were assigned to the frontotemporal subtype. Of the 57 presymptomatic mutation carriers, 48 (84%) were assigned to the normal-appearing group, 3 (5%) were assigned to the temporal subtype, and 6 (11%) were assigned to the frontotemporal subtype. Overall this gave a total of 33 subtypable (i.e., with detectable imaging abnormalities) mutation carriers, with a total of 23 individuals (68%) in the temporal subtype and 11 individuals (32%) in the frontotemporal subtype at baseline.

### Subtype Demographics

Table 1 shows the demographics of the normal-appearing group, temporal subtype, and frontotemporal subtype. There were significant differences in age at visit, proportion of symptomatic individuals, and estimated years from onset (EYO) among the 3 groups, but no differences in the proportion of men and women. The normal-appearing group was the youngest (mean age 38.3 ± 11.1 years), contained no symptomatic individuals, and had the longest estimated time until onset (average EYO of −15.0 ± 11.2 years). The temporal group was the oldest (mean age 59.0 ± 8.9 years), had the highest (87%) proportion of symptomatic individuals, and had the least estimated time until onset (average EYO of 4.8 ± 5.8 years, i.e., past onset). The frontotemporal group had a mean age of 47.7 ± 10.6 years, 45% symptomatic individuals, and an average EYO of −1.7 ± 8.7 years. SuStaIn stage was significantly correlated with EYO in the subtypable mutation carriers (*r* = 0.54, *p* ≤ 0.001, n = 34), with a similar correlation coefficient when analyzing each subtype individually (temporal: *r* = 0.49, *p* = 0.017, n = 23; frontotemporal: *r* = 0.51, *p* = 0.110, n = 11).

### Association Between MAPT Mutation and Subtype Assignment

We compared the subtype assignments (temporal vs frontotemporal) of individuals with different *MAPT* mutations, excluding the normal-appearing individuals assigned to very early SuStaIn stages at which there is low confidence in their subtype assignment. Table 2 compares the *MAPT* mutations of individuals assigned to each subtype. There was a one-to-one mapping between IVS10+16 and R406W mutations and assignment to the temporal subtype: 9/9 subtypable IVS10+16 mutation carriers and 7/7 subtypable R406W mutation carriers were assigned to the temporal subtype (*p* = 0.016 for IVS10+16 vs all other mutations and *p* = 0.040 for R406W vs all other mutations). There was a strong association between P301L mutations and assignment to the frontotemporal subtype (*p* < 0.001 vs all other mutations): 9/10 subtypable P301L mutation carriers were assigned to the frontotemporal subtype, with 1 subtypable P301L mutation carrier being assigned to the temporal subtype.

**Table 2:**
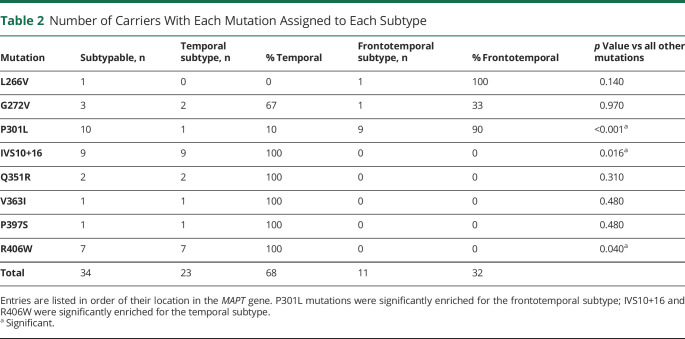
Number of Carriers With Each Mutation Assigned to Each Subtype

### Longitudinal Consistency of Subtypes

**Figure 2:**
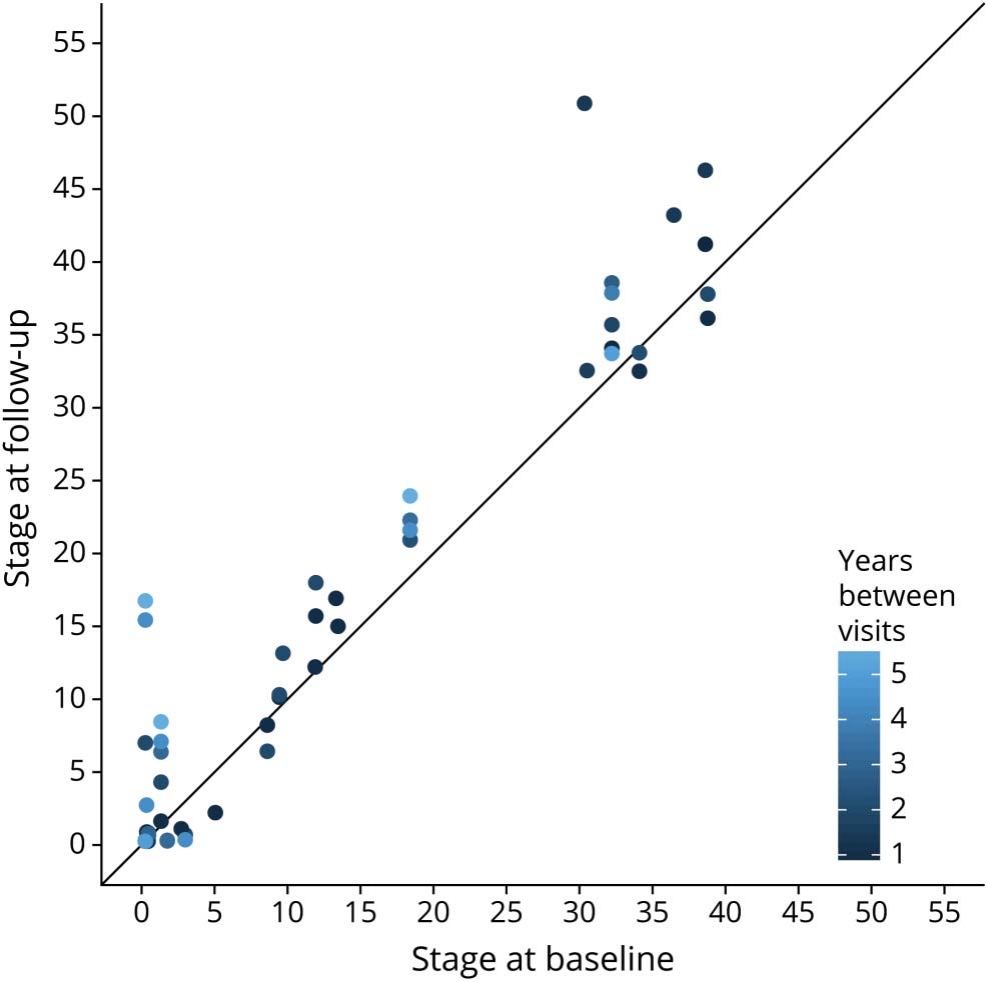
Stage Progression at Follow-Up Visits Each point represents an individual’s Subtype and Stage Inference (SuStaIn) stage at baseline and follow-up, with the color indicating the time between baseline and follow-up.

Fifty of the 82 *MAPT* mutation carriers had annual follow-up MRI scans at 1 or more time points, with a total of 92 follow-up scans available. Subtype assignments were generally stable at follow-up (Table 3), with subtype assignment remaining the same at 88 of the 92 follow-up visits. At the other 4 visits, 3 individuals progressed from the normal-appearing group to the temporal subtype, and 1 individual assigned to the frontotemporal subtype reverted to normal-appearing. No individuals changed from the temporal subtype to the frontotemporal subtype or vice versa. The individual who reverted from the frontotemporal subtype to normal-appearing at follow-up was only weakly assigned to the frontotemporal subtype at baseline, with a probability of 0.55 for frontotemporal and 0.38 for normal-appearing. Of the 3 individuals who progressed to the temporal subtype, 2 had IVS10+16 mutations and 1 had a rare mutation (undisclosed to avoid unblinding of genetic status). All 3 individuals were presymptomatic at baseline and remained presymptomatic at all available follow-up visits. Figure 2 shows the SuStaIn stages of individuals at follow-up compared to baseline. As expected, most individuals either progressed in stage or remained at the same stage at follow-up (i.e., are on or above the line y = x).

**Table 3:**
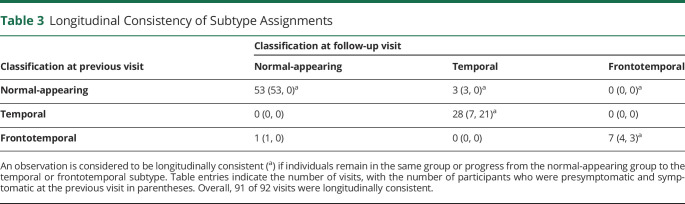
Longitudinal Consistency of Subtype Assignments

### Conversion From Presymptomatic to Symptomatic Stage

Two individuals converted from being presymptomatic to symptomatic within the current observational period of the study, both of whom were identified by SuStaIn as abnormal at baseline (i.e., were assigned to a subtype rather than to the normal-appearing group). Although both individuals had G272V mutations, 1 was assigned to the temporal subtype and the other to the frontotemporal subtype. Each individual had 1 available follow-up visit at which their respective subtype assignments remained the same.

### Neuropsychological Profile of Subtypes

Table 4 shows the relationship between neuropsychological test scores and SuStaIn subtype and stage across all subtypable carriers (presymptomatic and symptomatic), accounting for age and sex. eTable 2 (doi.org/10.5061/dryad.rxwdbrv83) reports the mean and median test scores in symptomatic carriers assigned to each subtype. Performance on the Digit Span forward and Block Design tasks was worse in the frontotemporal subtype but unrelated to SuStaIn stage, suggesting that performance on these tests has a stronger decline with disease progression in the frontotemporal subtype. Performance on the Boston Naming Test and both category and phonemic fluency tests was related to SuStaIn stage but not SuStaIn subtype, suggesting that these tests decline with disease progression in both subtypes. Performance on the TMT A and B and Digit Symbol tasks was worse in the frontotemporal subtype and related to SuStaIn stage, suggesting that these scores decline with disease progression in both subtypes but the overall scores are worse in the frontotemporal subtype. The associations between SuStaIn subtype and scores on the Digit Span forward and Block Design tests and SuStaIn stage and number of errors on the TMT A and B survived Bonferroni correction for multiple comparisons. In eTable 2, we further report group comparisons of test scores in symptomatic mutation carriers between subtypes, without correction for SuStaIn stage, age, or sex. Among symptomatic carriers, the Digit Span forward score remains significantly different between the temporal and frontotemporal subtype (*p* = 0.009) without correcting for confounders.

**Table 4:**
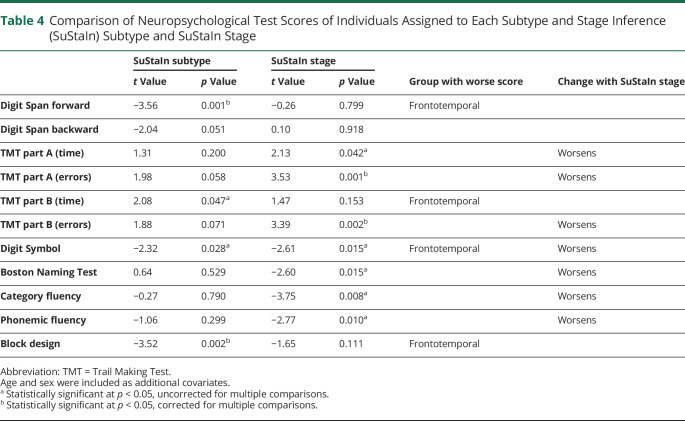
Comparison of Neuropsychological Test Scores of Individuals Assigned to Each Subtype and Stage Inference (SuStaIn) Subtype and SuStaIn Stage

### Clinical Characteristics of Subtypes

Table 5 shows the relationship between neuropsychological test scores and SuStaIn subtype and stage across all subtypable carriers (presymptomatic and symptomatic), accounting for age and sex. eTable 3 (doi.org/10.5061/dryad.rxwdbrv83) reports the mean and median scores in symptomatic carriers assigned to each subtype. Memory impairment score on the GENFI symptom scales (equivalent to the memory item on the CDR) and memory and orientation score on the CBI-R were worse in the temporal subtype but showed no relationship with SuStaIn stage, suggesting that memory decline is a feature of the temporal subtype only. Several clinical symptoms worsened with SuStaIn stage but were not related to SuStaIn subtype, suggesting that these are features of both subtypes. These symptoms were disinhibition, ritualistic or compulsive behavior, delusions, impaired grammar/syntax, dysgraphia, impaired functional communication, dysphagia on the GENFI symptom scales, and abnormal behavior and abnormal beliefs on the CBI-R. However, a large number of tests was performed, and consequently none survived Bonferroni correction for multiple comparisons. In eTable 3, we further report group comparisons of test scores in symptomatic mutation carriers between subtypes, without correction for SuStaIn stage, age, or sex. The memory impairment scores on both the GENFI symptom scales and the CBI-R remain significantly different (*p* = 0.003 and *p* = 0.007, respectively) between symptomatic carriers assigned to the temporal and frontotemporal subtype without correcting for confounders.

**Table 5:**
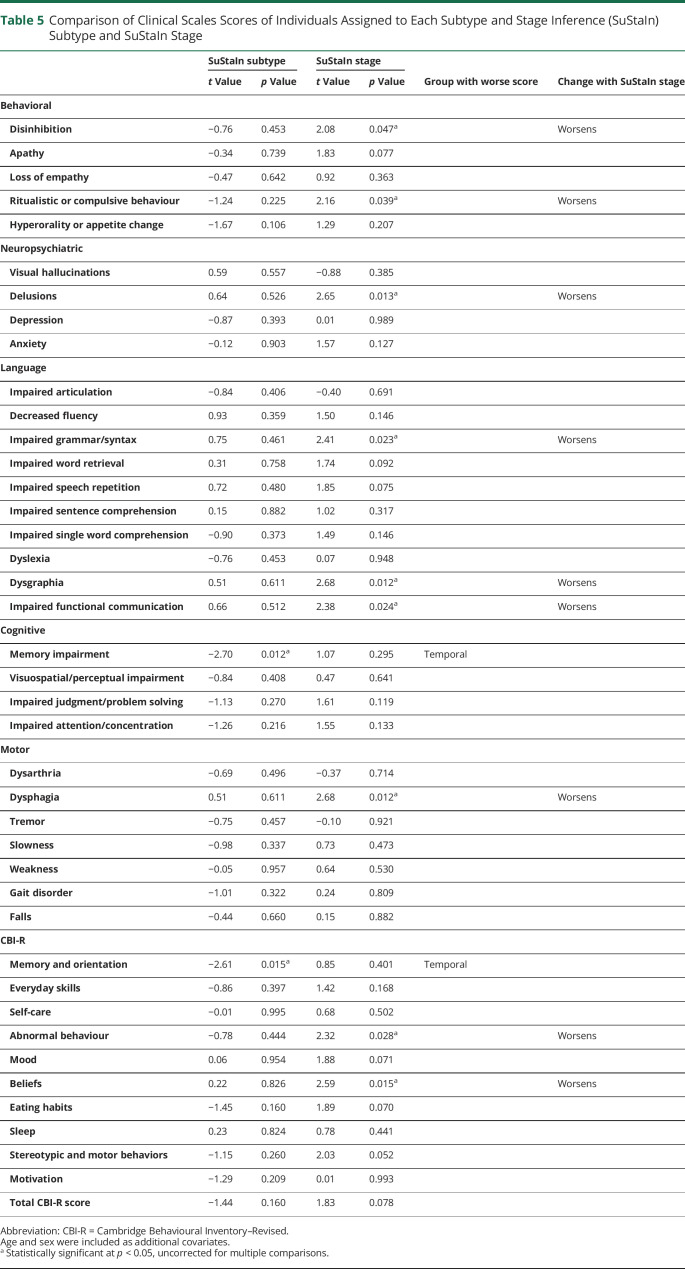
Comparison of Clinical Scales Scores of Individuals Assigned to Each Subtype and Stage Inference (SuStaIn) Subtype and SuStaIn Stage

## Discussion

We identified 2 distinct patterns of regional neurodegeneration in *MAPT* mutation carriers: a temporal subtype and a frontotemporal subtype. Each pattern was associated with different *MAPT* mutations and distinct cognitive and clinical symptoms. Our results provide new insights into the progression of tau pathology in *MAPT* mutations while also having potential utility for patient stratification.

The temporal and frontotemporal progression patterns identified by SuStaIn demonstrate that there are both common and distinct features between the 2 subtypes. Both subtypes have early volume loss in the anterior insula and lateral temporal lobe, but in the early stages of the temporal subtype, this atrophy is more widespread across other temporal lobe regions, including the hippocampus and amygdala, as well as the posterior insula, while in the early stages of the frontotemporal subtype there is additional atrophy in frontal regions. Our findings are broadly in agreement with the patterns identified in the studies by Whitwell et al.^7^ and Chu et al.,^14^ but account for variability in disease stage across individuals and use a larger sample size. Using SuStaIn, we are able to automatically group the mutations and reconstruct the full progression of atrophy including very early stages, which we can identify in presymptomatic individuals.

A higher proportion of presymptomatic mutation carriers was assigned to the frontotemporal subtype, and consequently the frontotemporal group was younger and further from onset than those assigned to the temporal subtype. This could indicate that the frontotemporal group tended to have less noticeable symptoms relative to the amount of neurodegeneration, either because they have greater cognitive reserve or because the symptoms are atypical compared to the expected set of symptoms in *MAPT* mutations. Alternatively, a higher proportion of presymptomatic individuals may indicate a longer presymptomatic phase among those assigned to the frontotemporal group.

SuStaIn identified one-to-one mapping between assignment to the temporal subtype and IVS10+16 and R406W mutations, demonstrating that these 2 mutations have a predictable atrophy pattern. This is in agreement with previous studies showing focal atrophy in the temporal lobe (particularly medially) in IVS10+16 and R406W mutation carriers.^7,15^ Q351R, V363I, and P397S mutations (found in either exon 13, similarly to R406W, or exon 12) also had a one-to-one mapping to the temporal subtype, but there were only a few individuals with these mutations in the study.

SuStaIn identified a strong relationship between P301L mutations and assignment to the frontotemporal subtype, with 9 out of 10 subtypable P301L mutation carriers being assigned to the frontotemporal subtype. This is in agreement with the results of Whitwell et al.^7^ and Chu et al.,^14^ who also identified P301L mutation carriers as having a different atrophy pattern vs those with intronic mutations. Interestingly, individuals assigned to the frontotemporal subtype all had mutations occurring earlier in the *MAPT* gene (L266V and G272V, both in exon 9, and P301L in exon 10), suggesting a possible relationship between location in the *MAPT* gene and atrophy pattern. It was also notable that no mutation had a one-to-one mapping to the frontotemporal subtype, whereas IVS10+16, Q351R, V363I, P397S, and R406W mutations all had a one-to-one mapping to the temporal subtype. This could be suggestive of multiple competing biological processes in L266V, G272V, and P301L mutations, producing either a temporal or a frontotemporal subtype. The phenotype produced by these mutations may be modified by additional genetic or environmental factors.^16^ Alternatively, the lack of a one-to-one mapping could simply be due to there being fewer samples from this group to train the SuStaIn algorithm on, making it more difficult to characterize the frontotemporal atrophy pattern.

The SuStaIn algorithm showed strong subtyping and staging capabilities: the subtype assignments were longitudinally consistent at 91 of the 92 follow-up visits, with 88 individuals remaining the same subtype and 3 individuals progressing from normal-appearing to subtypable. The individual who reverted from the frontotemporal subtype to normal-appearing at follow-up was only weakly assigned (probability of 0.55) to the frontotemporal subtype at baseline. Moreover, the 2 individuals who converted from being presymptomatic to symptomatic during the study were both subtypable (rather than normal-appearing) at baseline, suggesting that the SuStaIn algorithm might have utility for predicting symptom onset.

The frontotemporal group had worse performance on the Digit Span, TMT, Digit Symbol, and Block Design tasks compared to the temporal group, indicating greater deficits in tests that are likely to tap into executive function, consistent with the neuroanatomical findings of greater frontal lobe involvement. However, the temporal group had greater symptoms of memory impairment on the GENFI symptom scales and worse memory scores on the CBI-R. This is consistent with prior reports of episodic memory impairment in people with *MAPT* mutations,^17,18^ a feature that is generally unusual and atypical in FTD, but may well be a specific feature of certain *MAPT* mutations.

There are a number of limitations to our study and opportunities for future work. Subtyping was performed by simply assigning individuals to their most probable SuStaIn subtype given their imaging data; however, alternative methods for assigning subtypes using SuStaIn could be explored in the future, such as only subtyping individuals with a high probability of matching one of the subtypes. These types of approaches may be particularly beneficial when using SuStaIn in new populations with different demographics or unseen *MAPT* mutations. The statistical analysis of neuropsychological and clinical scores modeled SuStaIn subtype and stage simultaneously in order to pool data across the limited sample size, assuming that the test scores decline at the same rate within each subtype but have a different average value. There may be different rates of decline of test scores with stage within each subtype, which should be tested in future studies with larger sample sizes. While our study gathered the largest sample of *MAPT* mutation carriers to date, the numbers are still small and some mutations were absent from our study, such as the V337M mutation, and thus the subtypes may not be generalizable to individuals with these unseen mutations.

Overall, our results provide strong evidence of distinct patterns of atrophy in P301L mutations compared to IVS10+16 and R406W mutations in the largest sample of *MAPT* mutation carriers collected to date. We demonstrate that these distinct atrophy patterns produce different clinical phenotypes, with the temporal subtype being associated with impaired episodic memory and the frontotemporal subtype being associated with more executive dysfunction. The subtyping and staging information provided by the SuStaIn algorithm shows potential clinical utility for identifying individuals at risk of conversion and predicting their mutation, as well as for patient stratification in forthcoming therapeutic trials. Our results further demonstrate the power of the SuStaIn algorithm for identifying novel relationships between imaging phenotypes, genetics, and clinical presentation.

## Glossary

CBI-R: Cambridge Behavioural Inventory–revised
CDR: Clinical Dementia Rating
EYO: estimated years from onset
FTD: frontotemporal dementia
GENFI: Genetic FTD Initiative
GIF: geodesic information flow
SuStaIn: Subtype and Stage Inference
TMT: Trail Making Test
